# Hahnemann’s Homœopathic Formula

**Published:** 1884-12

**Authors:** Ad. Lippe

**Affiliations:** Philadelphia


### HAHNEMANN'S HOMŒOPATHIC FORMULA.

Dr. Ad. Lippe, Philadelphia.

The rejoinder of Dr. R. E. Dudgeon on the disputed propriety of substituting curentur for curantur has been read. We will now sum up the case and leave homœopathicians to enter a final decision.

Dr. Richard Hughes contended that the correct formula of Homœopathy, as Hahnemann himself used it, was similia similibus curentur. We took exception to and entered our protest against this new innovation. Dr. R. E. Dudgeon took up the defense of this innovation, to which we again replied, and now are presented by Dr. Dudgeon with a rejoinder. As Dr. Dudgeon says, "*I don't think there is any important difference of meaning between the one phrase or the other*," we again entirely differ with him. Whoever read the paragraphs 53 to 56 of Hahnemann's *Organon of the Healing Art*, and who also accepted the inductive method of Hahnemann, must arrive at the conclusion that the Law of the Similars and the formula expressing it, "similia similibus curantur," are mandatory, while curentur impliedly admits that while cures *may* be performed under the law of the similars there also may exist other applicable laws. Dr. Dudgeon *asserts* that Hahnemann always wrote curentur, and never curantur. The burden of the proof of this assertion rests with the other side. The question has been discussed before, and it has been found that the curentur *once* found in Hahnemann's writings was owing to the oversight of the proofreader, and if my memory, which has always been very good, does not betray me, it was the late Dr. Constantine Hering who showed the absurdity of substituting curentur for curantur.

The *British Journal of Homœopathy* did accept the formula, similia similibus curantur, from its inception up to the present day. This is no vague assertion; the documentary evidence is before us—*vide* Vol. I of that journal, published in 1843, and on page vii of the Introduction we find the following passage: "Our pages will be open for the expression of every shade of opinion, provided that the principle similia similibus curantur be fully admitted by the writers"! And now, in 1884, the discovery is made by Dr. Richard Hughes that curentur is right and curantur all wrong; and then comes to his assistance Dr. Dudgeon, who asserts that Hahnemann always wrote curentur! Has the British journal lived in error for over forty long years? Strange things have happened since the *British Journal of Hom-*

*œopathy* started, loyal to our school, its principle and rules, loyal to Hahnemann. That same Dr. Hughes discovered that Hahnemann's notes to Belladonna were erroneous because he himself and his Parisian friend could not find them. It was then shown him that his inability to find the original quotations was no proof of their non existence, and finally he was compelled to make an apology, as they were found, and the correctness of the never-before doubted, conscientious statements of Samuel Hahnemann was reasserted. Then came the assertion from the same quarter that the senility of that great philosopher made his late discoveries and his late writings of doubtful value. All of these discreditable statements were published in that primitively loyal *British Journal of Homœopathy*. Every individual who persistently follows a course of departures from long-acknowledged laws and rules of practice must have "*a motive*," and all who become his aiders and abettors must have a like motive. The motive has become apparent, as the departures increased, till at last the final blow was struck, and Homœopathy, with its ancient, time-honored formula, similia similibus curantur, was to be supplanted by illogical eclecticism, and its formula would properly read simila similibus curentur, so that the people might believe that the eclectics were homœopathists of an advanced type. These same men ask for recognition by the "regulars" and denounce openly Hahnemann and his followers.

Dr. Dudgeon may claim that there is no material difference between curantur and curentur; it is not likely that any loyal homœopathician will accept it as a fact that they are phrases only, almost synonymes. We have said enough. We shall, during the rest of our natural lifetime, hold fast to the old formula, and we wish Drs. Hughes, Dudgeon & Co. a happy time under their newly discovered formula. Success creates success, and if a superior success follows the practice of eclecticism than followed Hahnemann's methods and that of his faithful followers, then the eclectics will carry the day, the people will indorse them and condemn the faithful followers of Hahnemann; but if the eclectics fail to cure while the faithful followers of Hahnemann *do* cure, the verdict of the most interested part of the community will judge very differently. Frequently repeated prayers, directed to the eclectic wing of homœopathists, to show *one single curable case* treated under a strict application of the similars (similia similibus curantur) which was not cured and was afterward cured under another law of cure. *Then*, and not till then, will we join the followers of the newly discovered heresy of *curentur.*

